# Effects of *Lonicera japonica* extract on performance, blood biomarkers of inflammation and oxidative stress during perinatal period in dairy cows

**DOI:** 10.5713/ajas.19.0388

**Published:** 2019-10-21

**Authors:** Yiguang Zhao, Zhiwen Tang, Xuemei Nan, Fuyu Sun, Linshu Jiang, Benhai Xiong

**Affiliations:** 1 State Key Laboratory of Animal Nutrition, Institute of Animal Sciences, Chinese Academy of Agricultural Sciences, Beijing 100193, China; 2 Beijing Key Laboratory for Dairy Cow Nutrition, Beijing University of Agriculture, Beijing 102206, China

**Keywords:** Blood Biomarkers, Dairy Cows, Lactation, *Lonicera Japonica*, Perinatal Period

## Abstract

**Objective:**

An experiment was conducted to evaluate the effects of *Lonicera japonica* extract (LJE) on milk production, rumen fermentation and blood biomarkers of energy metabolism, inflammation and oxidative stress during the perinatal period of Holstein dairy cows.

**Methods:**

Eighteen Holstein dairy cows were used in a complete randomized design experiment with 3 dietary treatments and 6 cows per treatment. All cows received the same basal total mixed ration (TMR) including a prepartal diet (1.35 Mcal of net energy for lactation [NE_L_]/kg of dry matter [DM], 13.23% crude protein [CP]) from −60 d to calving and a postpartal diet (1.61 Mcal of NE_L_/kg of DM, 17.39% CP) from calving to 30 days in milk (DIM). The 3 dietary treatments were TMR supplemented with LJE at 0 (control), 1 and 2 g/kg DM, respectively. LJE was offered from 21 d before calving to 30 DIM. Dry matter intake (DMI) and milk production were measured daily after calving. Milk and rumen fluid samples were collected on 29 and 30 d after calving. On −10, 4, 14, and 30 d relative to calving, blood samples were collected to analyze the biomarkers of energy metabolism, inflammation and oxidative stress.

**Results:**

Compared with control diet, LJE supplementation at 1 and 2 g/kg DM increased DMI, milk yield and reduced milk somatic cell count. LJE supplementation also decreased the concentrations of blood biomarkers of pro-inflammation (interleukin-1β [IL-1β], IL-6, and haptoglobin), energy metabolism (nonesterified fatty acid and β-hydroxybutyric acid) and oxidative stress (reactive oxygen metabolites), meanwhile increased the total antioxidant capacity and superoxide dismutase concentrations in blood. No differences were observed in rumen pH, volatile fatty acid, and ammonia-N (NH_3_-N) concentrations between LJE supplemented diets and the control diet.

**Conclusion:**

Supplementation with 1 and 2 g LJE/kg DM could increase DMI, improve lactation performance, and enhance anti-inflammatory and antioxidant capacities of dairy cows during perinatal period.

## INTRODUCTION

Dairy cows experience substantial metabolic and physiological changes during the perinatal period due to their negative energy balance [1], inflammation [2], oxidative stress [3], and immune dysfunction [4]. For example, failure to meet the energy requirements for fetal growth and lactation during this period may lead to metabolic disorders such as ketosis and fatty liver and compromised antioxidant mechanisms [5,6]. Polymorphonuclear neutrophilic leukocyte function was compromised during the transition period and may lead to peripartum mastitis and metritis [7]. Oxidative stress is induced by the increased production of free radicals and reactive oxygen species and consequently results in a decrease in antioxidant defense. Oxidative stress also often leads to damage of biologic macromolecules and dysregulation of metabolism and physiology [8], which further results in the imbalance of immune function and inflammatory responses during the transition period [9].

*Lonicera japonica*, also known as Japanese honeysuckle or golden-and-silver honeysuckle, is a traditional folk medicine and usually used to treat common colds, fevers, enteritis, pain, and swellings in East Asian countries including China, Japan and Korea. More than 140 compounds have been isolated and identified from *Lonicera japonica*, including organic acids, flavonoids, iridoid glycosides and saponins etc. [10]. There has been more and more interest in *Lonicera japonica* extract (LJE) because of its unique anti-inflammatory [11,12], antioxidative [13] and hepatoprotective activities [14]. However, the effects of LJE on performance and health of dairy cows during transition period has rarely been reported. Therefore, the objectives of this study were to evaluate the effects of LJE on milk production, rumen fermentation and blood biomarkers of energy metabolism, inflammation and oxidative stress during the peripartal periods of Holstein dairy cows.

## MATERIALS AND METHODS

All experimental procedures for this study were approved by the Animal Care and Use Committee of the Institute of Animal Sciences, Chinese Academy of Agricultural Sciences (IAS2018-1).

### Animal management

Animals were fed twice daily at 0700 h and 1800 h during the experimental period *ad libitum*. Dry cows were housed in a ventilated free stall barn until 5 d before expected parturition date, and then they were moved to individual maternity barn with straw bedding until calving. The cows stayed in the maternity barn for 5 to 9 d. After parturition, they were transferred to a lactation barn and milked at 0700 h, 1300 h, and 1900 h daily.

### Experimental design and dietary treatments

Eighteen Holstein dairy cows were used in a complete randomized design experiment with 3 dietary treatments (n = 6). The cows in each group were balanced according to their parity (3.3±1.2), body condition score (3.48±0.07), initial body weight (759±13 kg) and expected calving date. All cows received the same basal total mixed ration (TMR) including a prepartal diet (1.35 Mcal of net energy for lactation [NE_L_]/kg of dry matter [DM], 13.23% crude protein [CP]) from −60 d to calving and a postpartal diet (1.61 Mcal of NE_L_/kg of DM, 17.39% CP) from calving to 30 days in milk (DIM), which were formulated according to NRC [15] to meet or exceed the nutrition requirements. Diet ingredients and chemical composition are shown in Table 1. *Lonicera japonica* extract (LJE) was added from 21 d before expected calving date until 30 DIM. The 3 dietary treatments were basal TMR supplemented with LJE powder at 0 (control), 1 and 2 g/kg DM, respectively. The ethanol extract of *lonicera japonica* was purchased from a commercial supplier (Zhongxing Biotechnology Co. Ltd., Xian, China). Chlorogenic acid has been reported as the major organic acid and used as the chemical marker for the quality evaluation of LJE [16]. The content of chlorogenic acid was 10% in the LJE used in the current study analyzed by liquid chromatography (LC-20A, Shimadzu, Kyoto, Japan).

### Diets sampling and analysis

Automatic feeding equipment (Institute of Animal Sciences, Chinese Academy of Agricultural Sciences, Beijing, China, and NanShang Husbandry Science and Technology Co. Ltd., Henan, China) was used to record DMI after calving. Samples of each diet were collected twice per week, stored at −20°C and composited weekly for analysis of DM, CP, ether extract (EE), neutral detergent fiber (NDF), acid detergent fiber (ADF), ash, calcium (Ca), and phosphorus (P). The DM content was determined by oven drying at 105°C until the weight is constant (method 930.15, AOAC) [17]. The CP content was determined using Kjeldahl nitrogen analysis (method 945.16, AOAC) [17]. The EE content was determined using a Soxhlet extractor (method 945.16, AOAC) [17]. The NDF and ADF were analyzed using heat-stable amylase (Sigma no. A3306, Sigma Chemical Co., St. Louis, MO, USA) and sodium sulfite according to the procedure of Van Soest et al [18]. Ash was measured by combustion using a muffle furnace (method 942.05, AOAC) [17]. The colorimetric method was used for analysis of phosphorus (Spectrophotometer UV752N, Yoke Instrument Co. Ltd., Shanghai, China) and calcium was measured using atomic absorption spectrometry (PerkinElmer AAS800, Waltham, MA, USA).

### Blood sampling and analysis

Blood samples were collected from the coccygeal vessel before the morning feeding on −10 d relative to expected calving and on 4, 14, and 30 d after calving. Samples were collected into 10-mL vacutainer tubes (Kindly Enterprise Development Group Co., Ltd., Shanghai, China) containing either clot activator or sodium heparin for serum and plasma, respectively. Serum and plasma were harvested by centrifugation at 3,000 ×g for 15 min at 4°C and stored at −80°C for further analysis. Serum and plasma were analyzed using a clinical auto-analyzer (GF-D200, Shandong Caihong Analytical Instruments Co. Ltd., Shandong, China) for glucose, urea, β-hydroxybutyric acid (BHBA), nonesterified fatty acid (NEFA), aspartate aminotransferase (AST), gamma-glutamyl transferase (GGT), cholesterol, total antioxidant capacity (T-AOC), superoxide dismutase (SOD), and glutathione peroxidase (GSH-Px). The concentrations of ceruloplasmin, haptoglobin, reactive oxygen metabolites (ROM), interleukin-1β (IL-1β) and IL-6 were determined according to the procedures described by Jacometo et al [19] and absorbance was read with a microplate reader (Multiskan MK3, Thermo Labsystems, Philadelphia, PA, USA).

### Milk sampling and analysis

Milking facilities of Afimilk (Side-by-Side Parallel Stall Construction, Afimilk Ltd. Kibbutz Afikim, Israel) were applied to record milk production of each cow after calving. Milk samples were collected on 29 and 30 d after calving for composition analysis. Samples collected in the morning, midday and evening were mixed with a ratio of 4:3:3 and stored with preservative (potassium dichromate) at 4°C until analysis. Milk fat, protein, lactose and somatic cell count (SCC) contents were analyzed by a mid-infrared spectroscopy (Fossomatic 4000, Foss Electric A/S, Hillerød, Denmark). Milk fat and protein yields were calculated by milk fat and protein contents multiplied milk yield, respectively and energy-corrected milk (ECM) and 4% fat-corrected milk (FCM) were calculated according to the equations: ECM = 0.327×milk (kg/d)+12.95×fat (kg/d) +7.65×protein (kg/d) and 4% FCM = 0.4×milk (kg/d)+15×fat (kg/d), respectively (NRC, 2001) [15].

### Ruminal fluid sampling and analysis

Rumen contents were collected at 2 h post-feeding using a stomach-tube (gastric lavage flexible pipe hose) on 29 and 30 d after calving. To reduce the contamination of saliva, the first 250 mL rumen fluid was discarded. Rumen contents were strained through 4 layers of cheesecloth with a mesh size of 250 μm and immediately determined pH with a portable pH meter (370 model pH meter, Jenway, London, UK). Each 10 mL of filtered rumen fluid of samples was mixed with 2 mL of 250 g/L metaphosphoric acid and stored at −20°C for volatile fatty acid (VFA) and NH_3_-N determination. Individual and total VFA (TVFA) were analyzed by gas chromatograph (GC-2010, Shimadzu, Japan) and NH_3_-N content was determined by phenol-hypochlorite method using a microplate reader (Multiskan MK3, Thermo Labsystems, USA).

### Statistical analysis

Data were analyzed as a completely randomized design using analysis of variance for evaluation of the effects of LJE on animal performance, rumen fermentation parameters and blood biomarkers of energy metabolism, inflammation and oxidative stress. The statistical program used in the present study was SAS 9.4 (SAS Institute Inc., Cary, NC, USA) with probability levels of p≤0.05 for significance and 0.05<p≤0.10 for tendency in treatments.

## RESULTS

### Milk production and composition

As shown in Table 2, LJE supplementation at 1 and 2 g/kg DM both had greater DMI (p<0.05), milk yield (p<0.05), milk fat yield (p<0.01), milk protein yield (p<0.01), 4% FCM (p< 0.05) and ECM (p<0.05) and less milk SCC (p<0.05) than the control diet. Moreover, LJE supplementation at 1 g/kg DM had better DMI and milk yield than 2 g/kg DM group (p<0.05). However, there was no significant effect of LJE supplementation on milk protein, fat or lactose content.

### Blood biomarkers

The effects of LJE supplementation on blood biomarkers are shown in Table 3. The NEFA, BHBA, IL-1β, IL-6, ROM and haptoglobin concentrations were lower (p<0.05), meanwhile the T-AOC and SOD concentrations were higher (p<0.05) in LJE supplementation groups than those in the control group. Moreover, the 1 g/kg DM LJE supplementation group had greater T-AOC and less ROM and haptoglobin concentrations in the blood than those in the 2 g/kg DM LJE group. There was no significant effect of LJE supplementation on the concentrations of glucose, cholesterol, urea, GGT, AST, GSH-Px, albumin and ceruloplasmin in blood.

### Rumen fermentation parameters

The effects of LJE supplementation on rumen pH, NH_3_-N and VFA concentrations are shown in Table 4. No differences between LJE supplementation diets and the control diet were observed in any variables of pH, NH_3_-N, acetate, propionate, butyrate, isovalerate, valerate, and TVFA concentrations, and the ratio of acetate/propionate. However, TVFA concentration was tended (p = 0.07) to increase in the LJE supplementation groups compared with the control group.

## DISCUSSION

### Milk production and composition and dry matter intake

During the perinatal period, negative energy balance occurs in dairy cows due to reduced DMI and increased nutrient requirements, which has been reported associated with increased disease susceptibility. In the present study, we found that LJE supplementation at doses of 1 and 2 g/kg DM increased DMI of the dairy cows. Doepel et al [20] reported that inflammatory response reduced DMI during the peripartum period of dairy cows. In the current study, LJE supplementation significantly decreased blood inflammatory cytokine concentrations (e.g. IL-6 and IL-1β, Table 3), indicating that inflammation was possibly eased. Hence, LJE supplementation increased DMI and consequently increased the milk yield. However, the milk fat and protein concentrations were not different between the LJE supplementation and the control. Therefore, the milk fat and protein yields, 4% FCM and ECM were not affected by LJE supplementation either. Somatic cell count is the indicator of infection within the udder, and SCC >200,000 cells/mL is considered as being infected by bacteria [21]. In the current study, SCC values of all groups were below 200,000 cells/mL, and the milk SCC in the cows offered LJE supplementation diets were even lower than those offered the control diet. This indicated that adding LJE in diets could effectively reduce the risk of udder infection during the prepartum period.

### Biomarkers of energy metabolism

To balance the negative energy during the transition period, dairy cows mobilize adipose tissue to meet energy requirements [2]. However, excessive lipid mobilization leads to increased blood and liver NEFA concentrations and consequently results in proinflammatory periparturient diseases and fatty liver [5]. Furthermore, as a major ketone body, BHBA can be produced by NEFA oxidation in the liver, and the extreme accumulation of BHBA may lead to ketosis [6]. In the present study, supplementation of LJE at doses of 1 and 2 g/kg DM reduced both NEFA and BHBA concentrations in blood. Previous study has demonstrated that supplementation with chlorogenic acid regulated energy metabolism and decreased serum BHBA concentration in rats [22]. Moreover, LJE supplementation increased the intake of the cows in the current study, therefore reduced the concentrations of NEFA and BHBA by alleviating the mobilization of adipose tissue. These results indicated that LJE supplementation at 1 and 2 g/kg DM could possibly improve the negative energy balance of dairy cows during the transition period.

### Biomarkers of oxidative stress

The imbalance between the production of ROM and antioxidants leads to oxidative stress [23]. The ROM is one of the most abundant free radicals in animals, and has been widely used as a biomarker for oxidative stress [24]. On the other hand, T-AOC is an important indicator in assessing concentrations of antioxidants which reflects the ability of reducing free radicals in the animal body [25]. Furthermore, SOD is recognized as a critical antioxidant enzyme in eliminating free radicals [26]. LJE has been reported possessing antioxidant activity in which chlorogenic acid in LJE is confirmed as the main effective ingredient [13]. Palíková et al [14] also found that the phenolic components isolated from *Lonicera japonica* alleviated oxidative damage of rat liver microsome and human umbilical vein endothelial cells. In the present study, LJE supplementation at doses of 1 and 2 g/kg DM decreased ROM and increased T-AOC and SOD concentrations. This may be attributed to the chlorogenic acid in LJE which has been approved with appreciable antioxidant potential in eliminating hydroxyl radicals and superoxide anion radical [16]. Therefore, LJE supplementation could improve the antioxidant capacity of the dairy cows during the perinatal period.

### Biomarkers of inflammation

Proinflammatory cytokines including TNF-α, IL-1β, IL-6, and IL-8 are closely linked to the severity of coliform mastitis during the periparturient period [26], which could effectively stimulate neutrophils to produce prostaglandins and leukotrienes, and sequentially increase the local inflammatory reactions [27]. Zhou et al [24] demonstrated that proinflammatory cytokines were increased during the transition period due to dramatic metabolic changes. In the current study, IL-1β and IL-6 concentrations were lower in the cows offered LJE supplementation diets than those fed the control diet. Park et al [11] found that the polyphenol components isolated from *Lonicera japonica* had anti-inflammatory effect on mouse peritoneal macrophage cells. Kang et al [12] also reported that luteolin from the *Lonicera japonica* inhibited inflammatory cytokines release by regulating nuclear factor-kappa B and mitogen-activated protein kinases pathways in human mast cell. As a major acute phase protein, haptoglobin has been used as a diagnostic biomarker due to its quick increase during the acute-phase response. Acute phase proteins are mainly produced by hepatocytes activated by the proinflammatory cytokines (e.g., IL-1β and IL-6) [28]. In the current study, LJE supplementation decreased the blood haptoglobin concentration, which, on the other hand, agreed with the decreased concentrations of IL-1β and IL-6 as previously described. These results indicated that LJE could contribute to reducing the risk of inflammation during the periparturient period.

### Rumen fermentation parameters

Supplementation of LJE tended to increase TVFA concentration in the current study. This could be explained by that LJE supplementation increased DMI, therefore providing more fermentation substrates for the rumen microorganisms. However, LJE had no significant effect on pH, NH_3_-N and individual VFA concentrations. This may be due to the microorganism ecosystem gradually adapted to the LJE addition in the rumen owing to long-term (from 21 d before calving to 30 DIM) feeding [29]. It has been well documented that rumen individual VFA concentrations are closely corelated with the milk composition [30]. Similarly, the unaffected VFA composition could possibly explain the corresponding stable milk ingredients across the three dietary treatments in the current study.

## CONCLUSION

Overall, supplementation with 1 and 2 g LJE/kg DM during the periparturient period could increase DMI and milk production and tend to increase TVFA concentration. By analyzing the blood biomarkers of lipid mobilization, oxidative stress and inflammation, LJE supplementation could also contribute to improving the capabilities of anti-inflammation and antioxidant of dairy cows. Therefore, LJE could be a potential natural plant substitute for the antibiotics to prevent metabolic diseases during perinatal period and improve animal performance. However, its anti-inflammation and antioxidant mechanisms still need to be further investigated.

## Figures and Tables

**Table 1:** Ingredients and nutrient composition of diets during dry period (−60 d to calving) and early lactation (calving to 30 d) (% of DM, unless otherwise stated)

Items	Dry period	Early lactation
Ingredients		
Corn silage	20.0	29.7
Oat hay	60.1	6.2
Alfalfa haylage	1.7	8.5
Corn ground	3.6	18.1
Extruded soybeans	-	2.6
Soybean meal	6.5	9.0
Rapeseed meal	1.0	4.5
Cottonseed meal	0.9	2.6
Cottonseed	-	2.7
Corn hull	5.1	9.6
Fat powder	-	1.2
Calcium salts of fatty acids1)	-	1.1
Mineral/vitamin premix A2)	1.1	-
Mineral/vitamin premix B3)	-	4.2
Nutrient composition		
NE_L_4) (Mcal/ kg DM)	1.35	1.61
CP	13.23	17.39
Ether extract	3.15	4.23
NDF	50.91	31.53
ADF	31.37	18.49
Ash	6.52	5.74
NFC5)	26.19	41.11
Calcium	0.45	0.85
Phosphorus	0.35	0.41

DM, dry matter; NE_L_, net energy for lactation; CP, crude protein; NDF, neutral detergent fiber; ADF, acid detergent fiber; NFC, nonfiber carbohydrate.

1) Calcium salts of fatty acids contained 9% Ca and 82.5% fat.

2) Mineral/vitamin premix A contained 1,500 mg/kg Cu, 938 mg/kg Mn, 11% NaCl, 4,500 mg/kg Zn, 65 mg/kg Se, 120 mg/kg I, 50 mg/kg Co, 425,000 IU/kg vitamin A, 174,000 IU/kg vitamin D, and 4,500 IU/kg vitamin E.

3) Mineral/vitamin premix B contained 12% Ca, 11% NaCl, 1,000 mg/kg Zn, 250 mg/kg Cu, 955 mg/kg Mn, 20 mg/kg Se, 98 mg/kg I, 40 mg/kg Co, 125,000 IU/kg vitamin A, 19,000 IU/kg vitamin D, and 1,500 IU/kg vitamin E.

4) NE_L_ was estimated according to NRC (2001).

5) NFC = 100 − (% NDF + % CP + % ether extract + % ash) (NRC, 2001).

**Table 2:** Effects of *Lonicera japonica* extract supplementation on DMI and milk production and composition during the peripartal period in Holstein dairy cows (n = 6)

Items	CON	1 g/kg DM	2 g/kg DM	SEM	p-value
DMI (kg/d)	16.2^c^	17.0^a^	16.6^b^	0.47	0.04
Yield (kg/d)					
Milk	20.00^c^	22.40^a^	20.98^b^	0.305	0.03
Fat	0.72^b^	0.84^a^	0.80^a^	0.115	0.04
Protein	0.67^b^	0.75^a^	0.72^a^	0.065	<0.01
ECM1)	20.98^c^	23.84^a^	22.69^b^	0.411	0.02
4% FCM2)	18.80^c^	21.49^a^	20.39^b^	0.366	0.04
Milk composition (%, unless otherwise stated)					
Protein	3.36	3.34	3.42	0.056	0.76
Fat	3.61	3.72	3.81	0.151	0.11
Lactose	4.99	5.23	4.86	0.055	0.12
SCC (10^4^/mL)	12.38^a^	3.78^b^	3.66^b^	1.253	0.04

DMI, dry matter intake; DM, dry matter; SEM, standard error of the mean; ECM, energy-corrected milk; FCM, fat-corrected milk; SCC, somatic cell count.

1) ECM = 0.327×milk (kg/d)+12.95×fat (kg/d)+7.65×protein (kg/d).

2) 4% FCM = 0.4×milk (kg/d)+15×fat (kg/d).

a–c Means within the same row with same superscripts are not different (p>0.05).

**Table 3:** Effects of *Lonicera japonica* extract supplementation on blood biomarkers (on −10, 4, 14, and 30 d relative to calving) during the peripartal period in Holstein dairy cows (n = 6)

Items	CON	1 g/kg DM	2 g/kg DM	SEM	p-value
Energy metabolism and liver function					
Glucose (mmol/L)	3.66	3.51	3.68	0.119	0.73
Cholesterol (mmol/L)	3.12	3.77	3.47	0.179	0.14
Urea (mmol/L)	3.47	3.61	3.08	0.257	0.24
GGT (U/L)	13.47	15.64	12.64	0.738	0.57
AST (U/L)	65.00	93.29	63.91	5.577	0.22
NEFA (μmol/L)	94.33^a^	76.51^b^	72.64^b^	3.261	0.04
BHBA (mmol/L)	0.94^a^	0.75^b^	0.78^b^	0.046	<0.01
Oxidative stress					
T-AOC (U/mL)	13.39^c^	21.65^a^	18.71^b^	0.822	<0.01
GSH-Px (μmol/L)	57.63	61.35	60.97	2.678	0.83
SOD (U/mL)	40.65^c^	47.08^a^	46.24^ab^	0.889	0.04
ROM (ng/L)	410.16^a^	297.11^c^	339.11^b^	14.964	<0.01
Inflammation					
Haptoglobin (mg/L)	243.63^a^	169.22c	184.36^b^	8.393	<0.01
Albumin (g/L)	32.73	34.14	34.18	1.298	0.89
Ceruloplasmin (U/L)	8.37	7.46	7.29	0.378	0.21
IL-1β (pg/mL)	113.24^a^	84.75^c^	90.54^bc^	3.929	<0.01
IL-6 (pg/mL)	1360.8^a^	914.5^b^	890.3^b^	44.02	<0.01

DM, dry matter; SEM, standard error of the mean; GGT, gamma-glutamyl transferase; AST, aspartate aminotransferase; NEFA, nonesterified fatty acid; BHBA, β-hydroxybutyric acid; T-AOC, total antioxidant capacity; GSH-Px, glutathione peroxidase; SOD, superoxide dismutase; ROM, reactive oxygen metabolites; IL, interleukin.

a–c Means within the same row with same superscripts are not different (p>0.05).

**Table 4:** Effects of *Lonicera japonica* extract supplementation on rumen fermentation (on 29 and 30 d after calving) during the peripartal period in Holstein dairy cows (n = 6)

Items	CON	1 g/kg DM	2 g/kg DM	SEM	p-value
Rumen pH	6.42	6.41	6.44	0.042	0.57
Ammonia-N (mg/dL)	19	24	21	0.3	0.23
Rumen VFA (mmol/L)					
Acetate	57.92	56.98	57.13	1.029	0.13
Propionate	24.27	25.43	23.87	0.524	0.76
Isobutyrate	0.69	0.89	0.82	0.149	0.17
Butyrate	8.39	9.21	9.63	0.211	0.24
Isovalerate	1.40	1.94	1.39	0.093	0.11
Valerate	1.21	1.42	1.99	0.151	0.27
Total VFA	94.23	96.27	95.28	1.214	0.07
Acetate/propionate	2.39	2.24	2.39	0.176	0.94

DM, dry matter; SEM, standard error of the mean; VFA, volatile fatty acid.
